# Budgeting for a billion: applying health technology assessment (HTA) for universal health coverage in India

**DOI:** 10.1186/s12961-018-0378-x

**Published:** 2018-11-29

**Authors:** Saudamini Vishwanath Dabak, Songyot Pilasant, Abha Mehndiratta, Laura Emily Downey, Francoise Cluzeau, Kalipso Chalkidou, Alia Cynthia Gonzales Luz, Sitaporn Youngkong, Yot Teerawattananon

**Affiliations:** 1 Health Intervention and Technology Assessment Program (HITAP), Nonthaburi, Thailand; 2 Global Health and Development Group, Imperial College London, London, United Kingdom; 3 Center for Global Development, London, United Kingdom; 4 Department of Pharmacy, Faculty of Pharmacy, Mahidol University, Bangkok, Thailand; 5 National Health Foundation, Bangkok, Thailand; 6 Saw Swee Hock School of Public Health (SSHSPH), National University of Singapore (NUS), Singapore, Singapore

**Keywords:** Health technology assessment in India

## Abstract

**Background:**

India recently launched the largest universal health coverage scheme in the world to address the gaps in providing healthcare to its population. Health technology assessment (HTA) has been recognised as a tool for setting priorities as the government seeks to increase public health expenditure. This study aims to understand the current situation for healthcare decision-making in India and deliberate on the opportunities for introducing HTA in the country.

**Methods:**

A paper-based questionnaire, adapted from a survey developed by the International Decision Support Initiative (iDSI), was administered on the second day of the Topic Selection Workshop that was conducted as part of the HTA Awareness Raising Workshop held in New Delhi on 25–27 July, 2016. Participants were invited to respond to questions covering the need, demand and supply for HTA in their context as well as the role of their organisation vis-à-vis HTA. The response rate for the survey was about 68% with 41 participants having completed the survey.

**Results:**

Three quarters of the respondents (71%) stated that the government allocated healthcare resources on the basis of expert opinion. Most respondents indicated reimbursement of individual health technologies and designing a basic health benefit package (93% each) were important health policy areas while medical devices and screening programmes were cited as important technologies (98% and 92%, respectively). More than half of the respondents noted that relevant local data was either not available or was limited. Finally, technical capacity was seen as a strength and a constraint facing organisations.

**Conclusion:**

The findings from this study shed light on the current situation, the opportunities, including potential topics, and challenges in conducting HTA in India. There are limitations to the study and further studies may need to be conducted to inform the role that HTA will play in the design or implementation of universal health coverage in India.

**Electronic supplementary material:**

The online version of this article (10.1186/s12961-018-0378-x) contains supplementary material, which is available to authorized users.

## Background

India, one of the fastest growing economies in the world and home to over a billion people [1, 2], celebrated 70 years of its formation in 2017. The occasion offered the opportunity to reflect on the accomplishments as well as the way forward for the country. Healthcare is one of the frontiers in which the country has achieved considerable advances, with the government having committed to progressively achieve universal health coverage (UHC) in line with the aspirations of the Sustainable Development Goals [3, 4]. Over seven decades, India has made strides in improving its indicators on healthcare [5]; however, public expenditure on health remains low, with high out-of-pocket expenditure and substantial variation in health outcomes across the country [6, 7]. Recently, the Government of India unveiled what will be the largest health insurance scheme in the world, the Ayushman Bharat – Pradhan Mantri Jan Aarogya Yojana [8].

The momentum for prioritising healthcare spending has been building over the years. In response to the growing demand from its citizens and commitment to expand access to quality healthcare and achieve UHC, the Planning Commission, now NITI Aayog, set up a High-Level Expert Group on UHC to examine what this would entail. Among its recommendations, the High-Level Expert Group called for increasing government spending on healthcare as well as the development of a national health package offering essential services [9]. In an effort to advance evidence-based decision-making for healthcare, the Planning Commission placed the mandate for health technology assessment (HTA) under the Department of Health Research (DHR) in the Ministry of Health and Family Welfare, Government of India [10]. Accordingly, a Medical Technology Assessment Board was to be set up [11] and, over the last couple years, the DHR has developed a structure to introduce HTA in making resource allocations at the national level, coordinated by the HTA India Secretariat or, HTAIn [12, 13]. The growing acceptance of HTA in health policy was also signalled in the recent National Health Policy, which committed to developing an institutional framework to support its adoption [4].

HTA is a multi-disciplinary approach that takes economic, medical, social and ethical considerations into account in a systematic and transparent manner to inform policy [14]. HTA provides policy-makers with the tools to allocate resources for health explicitly rather than implicitly [15]. This approach has been particularly relevant in countries where UHC policies are in place and the government is a strategic purchaser, as in the United Kingdom and Thailand. In the United Kingdom, the National Institute for Health and Care Excellence (NICE) uses HTA to explicitly define which interventions and technologies can be covered by the National Health Service, whereas in Thailand, the Health Intervention and Technology Assessment Program (HITAP) uses HTA to generate evidence and support the decision-making process for the Universal Coverage Scheme and the National List of Essential Medicines [16, 17]. HTA is an internationally accepted approach for using evidence to prioritise investments in healthcare. With this in mind, at the Prince Mahidol Award Conference 2016, Ministers of Health and development partners, among others, endorsed the Bangkok Statement on Priority Setting for UHC [18].

Since 2009, the Global Health and Development team at Imperial College (formerly NICE International), has been working with counterparts in India to support the establishment of HTA at the state and national levels, including developing Standard Treatment Guidelines [19]. On July 25, 2016, a high-level HTA Awareness Raising Workshop, organised by DHR and the International Decision Support Initiative or iDSI (a network of priority-setting institutions including Global Health and Development and HITAP), was held in New Delhi with Ministers of State in attendance. As part of the HTA Awareness Raising Workshop, a Topic Selection Workshop was held on July 26–27, 2016, for a targeted group of participants.

In this paper, we report on the results of a survey conducted during the Topic Selection Workshop. The objectives of the survey were to understand the current situation for healthcare decision-making in India, to identify the potential users and generators of HTA in the country, to deliberate on the opportunities, including topics, and challenges for the development of HTA in India. Through this study, we found that, although HTA development is at a nascent stage in India, there is an appetite for it in the country, with various opportunities for conducting HTA. There are, however, challenges related to technical capacity and infrastructure for HTA that will have implications for the future of HTA in India. This paper adds to the literature on assessing the potential policy use of HTA in the context of LMICs and, while India’s path towards UHC may well be unique, the lessons learned in this large and diverse country will resonate with other countries with limited resources as they strive towards UHC.

## Methods

### Survey design

The questionnaire was adapted from the Situation Analysis of HTA Introduction at National Level developed by iDSI partners. This questionnaire was tested in Vietnam, Indonesia and Ghana. The questionnaire consisted of four parts with a total of 24 questions, namely (1) need for HTA (4 questions), (2) demand for HTA (4 questions), (3) supply for HTA (6 questions) and (4) role of the respondent’s organisation in HTA (9 questions). Participants were requested to apply a national, state, municipal or other perspective in accordance with their role in the health system when responding to questions to reflect the difference in roles and responsibilities related to healthcare at each level. The full questionnaire is available in Additional files 1 and 2.

### Sample

We intended to include the opinions of all workshop participants who were invited by DHR to the workshop, namely government officials managing public health programmes, leaders and senior researchers from public and not-for profit research organisations, academics from universities, and staff from private sector and civil society organisations. In total, we provided 60 questionnaires and received responses from 41 participants (68% response rate). The majority of the respondents represented public organisations, including research institutions (38%), followed by academia and autonomous public institutions (36%). The remainder of the respondents were from government, private sector, non-governmental organisations and other types of research organisations. The respondents came from seven different states or union territories; however, two-thirds of the respondents were based in northern India, while about a quarter came from southern India and the remaining from either eastern or western India. Almost half of the respondents (49%) identified their organisation as being a generator of HTA, whereas 39% saw their organisation as both a user and generator of HTA. One respondent viewed their organisation as being neither a generator nor user of HTA. Table 1 provides information on the profile of the sample.

**Table 1:** Sample information

General information	Number	% Total
Type of organisation		
Government (Ministry and Civil Service)	4	10%
Public Organisation (including autonomous, research institutions)	16	41%
Academic Institutions (including autonomous public institutes for higher education)	13	33%
Other (including private sector and non-governmental organisations)	6	15%
Total	39	100%
Region		
North	25	66%
South, East and West	13	34%
Total	38	100%
Level		
National	24	59%
State	7	17%
Both or other^a^	10	24%
Total	41	100%
Perceived role of own organisation in HTA		
Generator	20	49%
User	4	10%
Both or other^a^	17	41%
Total	41	100%

^a^Since number of respondents to “other” was less than six, these have been combined to ensure anonymity

### Survey administration

The paper-based survey was conducted at the end of the second day of the 3-day HTA Awareness Raising and Topic Selection Workshop to ensure that participants had acquired the basic knowledge of HTA covered on the first and second days. All respondents were asked to complete the survey independently.

### Data entry and analysis

A maker-checker approach was used for entering data into Microsoft Excel. The data was analysed using descriptive statistics. Content analysis was used for coding and categorising qualitative data. Respondents were categorised into groups for analytical purposes and to ensure confidentiality of their responses, smaller groups were combined so as to remove identifying information. The main themes were analysed across the survey questions and reported.

## Results

In terms of allocation of healthcare resources, most respondents indicated that resources were being allocated on the basis of expert opinion (71%), followed by the impact of interventions on health outcomes (56%). In addition, 15% of respondents said that political considerations played a role in deciding how resources on healthcare were spent. Almost all respondents stated that efficient allocation of health resources (95%) was either an important or very important attribute for allocating resources, followed by transparency in decision-making and equity (93% each).

As part of the survey, respondents were asked to identify potential users and generators of HTA. Over half the respondents saw the government (either the ministry or civil service) as the main user of HTA (56%), particularly at the national level (41%). Public organisations (including autonomous, research institutions), on the other hand, were seen as being the main generators of HTA (55%), especially at the national level (59%). From the respondent’s perspective, users were likely to place more emphasis on budget impact and social and ethical considerations while making decisions on healthcare compared to generators of HTA.

Two prominent policy areas of importance to respondents were reimbursement of individual health technologies and designing a basic health benefit package (93% each) while reform of provider payment systems and service delivery for health were cited as being important or very important by 85% of the respondents. Among health technologies, almost all respondents pointed to medical devices as being very important to them (98%), followed by screening programmes (92%) and vaccines (90%). When asked to propose topics and research questions for HTA, a third of the respondents proposed topics related to health promotion and disease prevention, which included topics such as preliminary point of care of breast cancer screening devices. On the other hand, 20% and 15% of the respondents suggested topics related to service delivery and diagnostics, respectively. These results are summarised in Table 2.

**Table 2:** Policy areas, technologies and research topic areas

	Number	% Total
Health policy areas for HTA (important or very important)^a^		
Registration of individual health technologies	26	65%
Reimbursement of individual health technologies	37	93%
Clinical guidelines or disease management pathways development	33	83%
Design of basic package of health benefits	38	93%
Service delivery for health	34	85%
Reform of provider payment systems	34	85%
Health technologies for HTA (important or very important)^a^		
Medicines	34	85%
Vaccines	35	90%
Medical devices	40	98%
Screening programmes	36	92%
Referral programmes	30	75%
Procedures by health professionals (e.g. surgeries)	33	83%
Public health programmes or initiatives	34	89%
Service delivery initiatives or incentives	30	83%
Topics proposed by respondents		
Diagnostics	6	15%
Drugs and devices	4	10%
Health promotion and disease prevention	13	33%
Health services	4	10%
Service delivery	8	20%
Other	5	13%
Total	40	100%

^a^ Percentage calculated on total number of respondents to each question

The survey offered insights on the infrastructure available to conduct HTA as well as the strengths and constraints faced by organisations operating in the HTA landscape in India. In terms of the availability of data that are important for generating HTA evidence, it was found that there is limited or no access to data that is crucial for HTA analysis, primarily pharmaceutical use, cost of service delivery, hospital level data and health outcomes. Data was noted to be most limited for health outcomes, with only 20% of the respondents indicating its availability. On infrastructure for HTA, 70% of respondents pointed to the existence of health management information systems, followed by methodological guidelines (46%) and institutional processes (41%). The top three training needs evinced by respondents for HTA generators were for costing of healthcare (90%), health economic evaluation and economic modelling (88%), and meta-analysis (85%). For users, topic selection for HTA and institutional processes were deemed necessary by at least 80% of respondents. Approximately 10% of the respondents also called for a more general training on the process of policy-making using evidence. Respondents saw human or technical capacity as a strength (47%), with a third of these respondents stating that they had experience or ability to conduct systematic reviews or reviews. Human or technical capacity was also viewed as a constraint (34%) by respondents for their organisations to use or conduct HTA. In addition, an organisation’s network and positioning was seen as a strength by respondents (18%), whereas issues related to finance (21%) and policy environment (14%) were seen as constraints.

## Discussion

This study highlights the potential use of HTA for policy decision-making for healthcare as well as the areas in need of improvement for a successful take-off of HTA in India. Respondents to the survey perceived HTA as a tool that could help change implicit use of evidence in the form of expert opinion to a more explicit use of evidence by incorporating safety, efficacy, cost effectiveness, budget impact, and social and ethical considerations. These criteria were perceived as being equally important by the respondents. A previous stakeholder analysis of academics and technical assistance bodies in India has also stated that India has an informal decision-making process, with decisions based on published evidence or expert opinion. The stakeholders reported that HTA could help in creating a system for better decision-making [20]. Further, given the secular trends in healthcare costs, the demographic profile of the country and growth in technology, Dang et al. [21] point to the role of HTA in bringing efficiencies to the health system. In Thailand, Chaikledkaew et al. [22] report on the potential use of HTA and the limitations in terms of the supply of HTA in the country. The results are very similar in terms of the areas for HTA that need to be addressed. This study had been conducted when HTA was in an early stage of development in Thailand and the results were used in planning for its development. The activities included development of HTA guidelines and infrastructure for costing and utility estimates, and these elements are now being used to conduct HTA and inform health policies in the country.

Our results illustrate the potential policy areas, technologies and topics for HTA in India. Respondents had a common idea of where HTA can be applied, such as reimbursement of individual technologies and design of benefits package, which is in line with the use of HTA in other countries. Kumar et al. [23] note that there is potential for application of HTA in areas such as national and state level health insurance schemes. In addition, more than 80% of respondents agreed that use of HTA for provider payment systems is important or very important, and this may warrant in-depth exploration. In terms of types of technologies, respondents prioritised medical devices over vaccines and medicines, which are the more traditional areas for application of HTA in other countries. In fact, the HTA India Secretariat has taken up the assessment of intraocular lenses as one of its first HTA studies [24]. Interestingly, respondents indicated similar levels of importance for screening and public health programmes as they did for vaccines and medicines, and this may be because many of the respondents are from the national level where the policy focus may be on these issues. This is not dissimilar to findings from the WHO survey report, where respondents from low-income countries reported population-level health interventions as the foremost indication for HTA and used it less often for decisions regarding medicines, medical devices or surgical interventions [23]. This is further confirmed by a study commissioned by EUnetHTA [25], which shows that HTA agencies are likely to assess pharmaceuticals, medical procedures and medical devices.

The government, at both the national and state levels, was identified as the dominant user of HTA. This finding is similar to the reported global use of HTA, wherein ministries of health or national health insurance bodies have been identified as the main initiators of HTAs [26, 27]. However, unlike in several countries where HTA is applied, the government is not a large player in the health sector, contributing to only a third of overall health spending in the country [2]. Further, the governance system for healthcare is complex given the federal structure of the country and multiple stakeholders that operate therein [23]; while the central government is responsible for supporting medical education, managing regulatory bodies and supporting states among other activities, health policies are decided and implemented at the state level. This has implications for linking HTA to policy; at the national and state levels, HTA may be used to define the benefits package of insurance schemes such as the Rashtriya Swasthya Bima Yojana, to be subsumed under the Ayushman Bharat – Pradhan Mantri Jan Aarogya Yojana scheme, that covers families below the poverty line as well as those working in the unorganised sector [28, 29] or the various insurance schemes at the state level [30, 31]. Additionally, vertical programmes on AIDS or Tuberculosis or the National Health Mission, which supports states to improve or maintain key health indicators, may be other users of HTA to enhance delivery of care. In addition to the government, the pharmaceutical industry, insurance companies and healthcare providers have been identified as relevant stakeholders in the process [32].

Public health research output from India has increased over the years, although the quality of this research is still wanting [33, 34]. Further, existing literature on economic evaluations in India has been found to be inadequate to feed into sound policy-making [35]. While investments in data related to healthcare indicators have been made over the years as reflected in the manual on health statistics [36], data for conducting HTA studies remains limited as shown by the results of this survey. This indicates the simultaneous need to invest in public health research and create or strengthen data systems on service use and cost, among others, which are necessary to support HTA analyses in India. Another area that requires attention is building technical capacity for HTA. Fellowship programmes on HTA have been conducted by the National Health Systems Resource Center and the Amrita Institute of Medical Sciences to build capacity in the field [37, 38]. Health economics courses, such as the ones at the Post Graduate Institute of Medical Education and Research [39] and the Tata Institute of Social Sciences [40], are a step in the right direction. It has been suggested that, given the depth of analytical skills required, methods such as meta-analysis for synthesising results from elsewhere and regional collaboration may be pursued to build country capacity in HTA at the initial stage [41]. DHR has taken steps in this regard and, over the past year, has designed an HTA system comprising 15 resource hubs and technical partners from across the country to contribute to national and state level priorities [24]. It has hosted capacity-building workshops, including a series of workshops aligned with the life cycle of HTA studies for the technical partners in collaboration with iDSI [42].

Concerns about country capacity and availability of infrastructure to produce high quality HTA evidence may have a bearing on how the HTA mechanism is structured in the country. Given that there is limited capacity for generating HTA evidence, the most feasible way would be to apply HTA for decision-making at the national level. On the one hand, HTA could be conducted at the state level to ensure local relevance, but there would be insufficient capacity to perform state-by-state HTA studies. On the other hand, India is a large country with significant variations between states and union territories and it would be very challenging to show that the HTA results are nationally representative and relevant to all constituencies, particularly as policy formulation and implementation occurs at the state level. Further, India has only recently embarked on its journey of using HTA to inform policy in the country and the technical approach used in HTA may seem formidable to the stakeholders involved. India can learn from other countries such as Thailand, which has demonstrated how using HTA for informing coverage decisions for health benefit packages enhances the legitimacy of policy decisions by increasing the transparency, inclusiveness and accountability of the process [43]. Evidence from the region also suggests that, while there are barriers such as silo-based decision-making processes and heavy reliance on experts, there are several enabling factors that can catalyse the use of HTA including political commitment and effective collaboration among agencies [27].

There are some limitations to the study. The small sample size of the survey participants limits the generalisability of the findings from this study. Another limitation is the type of participants surveyed. Selection bias and non-random sampling bias also impact the survey results. A large number of respondents represented the government or public organisations, including research institutions, academia and autonomous public institutions, and there was comparatively lesser representation from the private sector, non-governmental organisations and civil society organisations. In addition, the survey was completed by only two-thirds of the participants of the workshop and the profile of non-respondents has not been studied. There was also unequal geographic representation as respondents came from only 7 out of a total of 36 states and union territories combined and a majority of the respondents were based in northern India.

## Conclusion

This study highlights opportunities for conducting and using HTA in India as part of its universal healthcare policy. The findings suggest that HTA provides a meaningful tool to design public health services and estimate its budget for expanding access to healthcare for India’s billion people as the country seeks to implement UHC in the coming year.

## Additional files

Additional file 1: Questionnaire. Survey on health technology assessment in India. (DOCX 31 kb)

Additional file 2: Summary of results. Tables summarising results of the data collected through the survey. (XLSX 15 kb)

## Abbreviations

DHR: Department of Health Research
HITAP: Health Intervention and Technology Assessment Program
HTA: Health Technology Assessment
iDSI: International Decision Support Initiative
NICE: National Institute for Health and Care Excellence
UHC: Universal Health Coverage

## References

[1] World Economic Outlook (WEO) Update. Washington DC: The International Monetary Fund, 2017. January 16, 2017. Report No

[2] World Development Indicators. Washington DC: World Bank; 2016.

[3] United Nations (2015). Transforming Our World: The 2030 Agenda for Sustainable Development. United Nations.

[4] Ministry of Health and Family Welfare Government of India. National Health Policy 2017. 2017. http://164.100.158.44/showfile.php?lid=4275. Accessed 18 Nov 2018.

[5] DGoHS: Directorate General of Health Services (DGHS). Central Bureau of Health Intelligence, Directorate General of Health Services. National Health Profile 2015. New Delhi: Ministry of Health and Family Welfare, Government of India; 2015.

[6] Reddy KS (2015). India’s Aspirations for Universal Health Coverage. N Engl J Med.

[7] MoSaPI: Ministry of Statistics and Programme Implementation (MoSPI). National Sample Survey Office, Ministry of Statistics and Programme Implementation. Key Indicators of Social Consumption in India Health. New Delhi; 2015.

[8] Ayushman Bharat Scheme (2018). Health insurance details, how to apply and more. The Times of India.

[9] High Level Expert Group (2011). The Planning Commission India.

[10] Planning Commission India (2013). Twelfth Five Year Plan (2012–2017) Social Sectors.

[11] MoHaFW: Ministry of Health and Family Welfare (MoH&FW). Press Information Bureau, Ministry of Health and Family Welfare, Government of India. Medical Technology Assessment Board to Be Set Up. 2013. http://pib.nic.in/newsite/mbErel.aspx?relid=101329. Accessed 18 Nov 2018.

[12] Prinja S, Downey LE, Gauba VK, Swaminathan S (2018). Health technology assessment for policy making in India: current scenario and way forward. PharmacoEconomics - Open.

[13] Department of Health Research, Ministry of Health and Family Welfare, Government of India. http://www.dhr.gov.in/mtab. Accessed 18 Nov 2018.

[14] World Health Organization. HTA Definitions. http://www.who.int/health-technology-assessment/about/Defining/en/. Accessed 10 Mar 2017.

[15] Chalkidou K, Glassman A, Marten R, Vega J, Teerawattananon Y, Tritasavit N (2016). Priority-setting for achieving universal health coverage. Bull World Health Organ.

[16] Teerawattananon Y, Kingkaew P, Koopitakkajorn T, Youngkong S, Tritasavit N, Srisuwan P (2016). Development of a health screening package under the universal health coverage: the role of health technology assessment. Health Econ.

[17] Teerawattananon Y, Tritasavit N, Suchonwanich N, Kingkaew P (2014). The use of economic evaluation for guiding the pharmaceutical reimbursement list in Thailand. Zeitschrift fur Evidenz, Fortbildung und Qualitat im Gesundheitswesen.

[18] Bangkok Statement. Priority Setting for Universal Health Coverage. Bangkok: Prince Mahidol Award Conference (PMAC); 2016. http://www.pmaconference.mahidol.ac.th/index.php?option=com_content&view=article&id=758&Itemid=216. Accessed 18 Nov 2018.

[19] Mehndiratta A, Sharma S, Prakash N, Sankar J, Cluzeau F. Adapting clinical guidelines in India – a pragmatic approach. BMJ. 2017;359:j5147. 10.1136/bmj.j5147. 10.1136/bmj.j5147 PMC5691554 29150419

[20] Jain B, Hiligsmann M, Mathew JL (2014). Analysis of a small group of stakeholders regarding advancing health technology assessment in India. Value Health Reg Issues.

[21] Dang A, Likhar N, Alok U (2016). Importance of economic evaluation in health care: an indian perspective. Value Health Reg Issues.

[22] Chaikledkaew U, Lertpitakpong C, Teerawattananon Y, Thavorncharoensap M, Tangcharoensathien V (2009). The current capacity and future development of economic evaluation for policy decision-making: a survey among researchers and decision-makers in Thailand. Value Health.

[23] Kumar M, Taylor FC, Chokshi M, Ebrahim S, Gabbay J (2014). Health technology assessment in India: The potential for improved healthcare decision-making. Natl Med J India.

[24] Health Secretariat (2018). Health Technology Assessment in India - HTAIn e-Newsletter.

[25] Moharra M, Kubesch N, Estrada MD, Parada T, Cortés M, Espallargues M (2008). Survey report on HTA organisations.

[26] World Health Organization. 2015 Global Survey on Health Technology Assessment by National Authorities Main findings. Geneva: WHO; 2015.

[27] Chootipongchaivat S, Tritasavit N, Luz A, Teerawattananon Y, Tantivess S. Factors Conducive to the Development of Health Technology Assessment in Asia: Impacts and Policy Options. Manila: World Health Organization, Regional Office for the Western Pacific, 2015. http://www.searo.who.int/entity/asia_pacific_observatory/publications/policy_briefs/policy_brief_factors_conducive/en/. Accessed 18 Nov 2018.

[28] Rashtriya Swasthya Bima Yojana (RSBY). About the Scheme. http://www.rsby.gov.in/about_rsby.aspx. Accessed 6 Sep 2017.

[29] Admin NHP. Ayushman Bharat Yojana. 2018. https://www.nhp.gov.in/ayushman-bharat-yojana_pg. Accessed 18 Nov 2018.

[30] Prinja S, Chauhan AS, Karan A, Kaur G, Kumar R (2017). Impact of publicly financed health insurance schemes on healthcare utilization and financial risk protection in india: a systematic review. PLoS ONE.

[31] Jain N (2017). Role of Government-funded and Community-based Health Insurance Schemes in Moving toward Universal Health Coverage in India.

[32] Dang A, Vallish B (2016). Can health technology assessment (HTA) provide a solution to tackle the increasing health-care expenditure in India?. Indian J Public Health.

[33] Dandona L, Sivan YS, Jyothi MN, Bhaskar VU, Dandona R (2004). The lack of public health research output from India. BMC Public Health.

[34] Dandona L, Raban MZ, Guggilla RK, Bhatnagar A, Dandona R (2009). Trends of public health research output from India during 2001-2008. BMC Med.

[35] Prinja S, Chauhan AS, Angell B, Gupta I, Jan S (2015). A systematic review of the state of economic evaluation for health care in India. Applied Health Econ Health Policy.

[36] Manual on Health Statistics in India. Central Statistical Office MoSaPI. Government of India. New Delhi2015.

[37] 5th International Fellowship on Health Technology Assessment 16–19 March 2015, Mumbai. World Health Organization (WHO) Southeast Asia Regional Office; 2015. http://www.searo.who.int/india/mediacentre/events/2015/fellowship_HTA/en/. Accessed 23 Sep 2018.

[38] Fellowship on Health Technology Assessment in India (2012). Amrita Institute of Medical Sciences (AIMS).

[39] Post-Graduate Institute of Medical Education and Research (PGIMER) Chandigarh. About School of Public Health. 2017. http://www.healtheconomics.pgisph.in/about-pgi-school-of-public-health.html. Accessed 3 Sep 2017.

[40] Master of Public Health (Health Policy, Economics and Finance). Tata Institute of Social Sciences (TISS). https://admissions.tiss.edu/view/10/admissions/ma-admissions/master-of-public-health-mph-in-health-policy-econo/. Accessed 18 Nov 2018.

[41] Glassman A, Giedion U, Sakuma Y, Smith PC (2016). Defining a health benefits package: what are the necessary processes?. Health Syst Reform.

[42] First capacity building for HTA in India workshop kicks off in Delhi (2018). International Decision Support Initiative (iDSI).

[43] Mohara Adun, Youngkong Sitaporn, Velasco Román Pérez, Werayingyong Pitsaphun, Pachanee Kumaree, Prakongsai Phusit, Tantivess Sripen, Tangcharoensathien Viroj, Lertiendumrong Jongkol, Jongudomsuk Pongpisut, Teerawattananon Yot (2012). Using health technology assessment for informing coverage decisions in Thailand. Journal of Comparative Effectiveness Research.

