# Supplementary material for: Budgeting for a billion: applying health technology assessment (HTA) for universal health coverage in India
Source: Health Res Policy Syst. 2018 Nov 29;16:115. doi: 10.1186/s12961-018-0378-x (PMC6262968; doi:10.1186/s12961-018-0378-x)
Supplement: Supplementary file 1 — Questionnaire. Survey on health technology assessment in India. (DOCX 31 kb) [file 12961_2018_378_MOESM1_ESM.docx]

# About this survey:

Greetings!

You are receiving this survey as you are participating in the Health Technology Assessment (HTA) Awareness Raising Workshop (25-27 July, 2016) in New Delhi organized by the Ministry of Health and Family Welfare, Government of India along with NICE International, UK.

The Health Intervention and Technology Assessment Program (HITAP), Ministry of Public Health, Thailand, is leading the sessions on Topic Selection on 26-27 July, 2016. In an effort to make discussions as relevant as possible, we would like to invite you to take this survey to hear your thoughts on HTA in India. The results of this survey will be anonymous and will be presented on the last day of the workshop.

This questionnaire has been adapted from the "Situation Analysis of HTA Introduction at National Level" developed by HITAP and NICE International.

# Instructions for completing this survey:

This survey is divided into four parts:

Part I: Need for HTA in your context

Part II: Demand for HTA in your context

Part III: Supply for HTA in your context

Part IV: Role of your organization in HTA

When responding to each section, you may think of one context in India - national, state, municipal or other.

Your responses will be anonymous.

Thank you for participating in our survey. If you have any questions, please write to us at

[hiu@hitap.net](mailto:hiu@hitap.net)

# Part I: Need for HTA in your context

1. In your opinion, how are resources for healthcare are allocated by the government in your context? *(select all that apply)*

🞎 Impact on health outcomes 🞎 Expert opinion

🞎 Advocacy groups 🞎 Donor priorities

🞎 Others, please specify

………………………………………………………………………………………....…

1. HTA helps address several aspects of policy making in health. Please rate each of the attributes listed below in terms of their importance to you:

|  | NI^[[1]](#footnote-1)^ | SI | MI | I | VI | N/A |
| --- | --- | --- | --- | --- | --- | --- |
| Efficient allocation of health resources | 🞎 | 🞎 | 🞎 | 🞎 | 🞎 | 🞎 |
| Transparency in decision making | 🞎 | 🞎 | 🞎 | 🞎 | 🞎 | 🞎 |
| Impact on government budget | 🞎 | 🞎 | 🞎 | 🞎 | 🞎 | 🞎 |
| Equity | 🞎 | 🞎 | 🞎 | 🞎 | 🞎 | 🞎 |
| Financial protection | 🞎 | 🞎 | 🞎 | 🞎 | 🞎 | 🞎 |
| Improving quality of healthcare | 🞎 | 🞎 | 🞎 | 🞎 | 🞎 | 🞎 |
| Other, please specify ……………………… | 🞎 | 🞎 | 🞎 | 🞎 | 🞎 | 🞎 |
| Other, please specify ……………………… | 🞎 | 🞎 | 🞎 | 🞎 | 🞎 | 🞎 |
| Other, please specify ……………………… | 🞎 | 🞎 | 🞎 | 🞎 | 🞎 | 🞎 |

1. HTA can be used in different health policy areas to improve the evidence base for decision making. Please rate each of the policy areas listed below in terms of their importance to you:

|  | NI | SI | MI | I | VI | N/A |
| --- | --- | --- | --- | --- | --- | --- |
| Registration of individual health technologies | 🞎 | 🞎 | 🞎 | 🞎 | 🞎 | 🞎 |
| Reimbursement of individual health technologies | 🞎 | 🞎 | 🞎 | 🞎 | 🞎 | 🞎 |
| Clinical guidelines or disease management pathways development | 🞎 | 🞎 | 🞎 | 🞎 | 🞎 | 🞎 |
| Design of basic package of health benefits | 🞎 | 🞎 | 🞎 | 🞎 | 🞎 | 🞎 |
| Service delivery for Health | 🞎 | 🞎 | 🞎 | 🞎 | 🞎 | 🞎 |
| Reform of provider payment systems | 🞎 | 🞎 | 🞎 | 🞎 | 🞎 | 🞎 |
| Other, please specify ……………………… | 🞎 | 🞎 | 🞎 | 🞎 | 🞎 | 🞎 |
| Other, please specify ……………………… | 🞎 | 🞎 | 🞎 | 🞎 | 🞎 | 🞎 |
| Other, please specify ……………………… | 🞎 | 🞎 | 🞎 | 🞎 | 🞎 | 🞎 |

1. HTA can be used to assess different types of health technologies. Please rate each of the policy areas listed below in terms of their importance to you:

|  | NI | SI | MI | I | VI | N/A |
| --- | --- | --- | --- | --- | --- | --- |
| Medicines | 🞎 | 🞎 | 🞎 | 🞎 | 🞎 | 🞎 |
| Vaccines | 🞎 | 🞎 | 🞎 | 🞎 | 🞎 | 🞎 |
| Medical devices | 🞎 | 🞎 | 🞎 | 🞎 | 🞎 | 🞎 |
| Screening programs | 🞎 | 🞎 | 🞎 | 🞎 | 🞎 | 🞎 |
| Referral programs | 🞎 | 🞎 | 🞎 | 🞎 | 🞎 | 🞎 |
| Procedures by health professionals (e.g. surgeries) | 🞎 | 🞎 | 🞎 | 🞎 | 🞎 | 🞎 |
| Public health programs or initiatives | 🞎 | 🞎 | 🞎 | 🞎 | 🞎 | 🞎 |
| Service delivery initiatives or incentives | 🞎 | 🞎 | 🞎 | 🞎 | 🞎 | 🞎 |
| Other, please specify ……………………… | 🞎 | 🞎 | 🞎 | 🞎 | 🞎 | 🞎 |
| Other, please specify ……………………… | 🞎 | 🞎 | 🞎 | 🞎 | 🞎 | 🞎 |
| Other, please specify ……………………… | 🞎 | 🞎 | 🞎 | 🞎 | 🞎 | 🞎 |

# Part II: Demand for HTA in your context

1. Please list ONE organization that is a potential user of HTA in your context:

……………………………………………………..…………………………………………

1. At which level does this organization operate:

🞎 National 🞎 State

🞎 Both 🞎 Other, please specify ……………………………

1. For the HTA user you identified above, please rate how important you think this organization rates the following attributes:

|  | NI | SI | MI | I | VI | N/A |
| --- | --- | --- | --- | --- | --- | --- |
| Safety | 🞎 | 🞎 | 🞎 | 🞎 | 🞎 | 🞎 |
| Efficacy | 🞎 | 🞎 | 🞎 | 🞎 | 🞎 | 🞎 |
| Cost Effectiveness | 🞎 | 🞎 | 🞎 | 🞎 | 🞎 | 🞎 |
| Budget Impact | 🞎 | 🞎 | 🞎 | 🞎 | 🞎 | 🞎 |
| Social and ethical considerations | 🞎 | 🞎 | 🞎 | 🞎 | 🞎 | 🞎 |
| Other, please specify ……………………… | 🞎 | 🞎 | 🞎 | 🞎 | 🞎 | 🞎 |

1. Please identify training needs to improve HTA capacity of evidence users *(select all that apply)*:

🞎 Introduction and Application of HTA 🞎 Topic Selection Process for HTA

🞎 Overview of Health Economics 🞎 Institutional processes for HTA

🞎 Other, please specify

………………………………………………………………………………………………………………………………………………………………………………………………………………………………………………………………………..………………………

# Part III: Supply for HTA in your context

1. Please identify ONE organization that supplies or generates evidence to support health policy decisions in your context.

……………………………………………………..…………………………………………

1. At which level does this organization operate:

🞎 National 🞎 State

🞎 Both 🞎 Other, please specify ……………………………

1. For the HTA evidence generator you identified above, please rate how important you think this organization rates the following attributes:

|  | NI | SI | MI | I | VI | N/A |
| --- | --- | --- | --- | --- | --- | --- |
| Safety | 🞎 | 🞎 | 🞎 | 🞎 | 🞎 | 🞎 |
| Efficacy | 🞎 | 🞎 | 🞎 | 🞎 | 🞎 | 🞎 |
| Cost Effectiveness | 🞎 | 🞎 | 🞎 | 🞎 | 🞎 | 🞎 |
| Budget Impact | 🞎 | 🞎 | 🞎 | 🞎 | 🞎 | 🞎 |
| Social and ethical considerations | 🞎 | 🞎 | 🞎 | 🞎 | 🞎 | 🞎 |
| Other, please specify ……………………… | 🞎 | 🞎 | 🞎 | 🞎 | 🞎 | 🞎 |

1. Availability of local data to inform country-specific decisions is a key challenge of HTA. Please indicate the availability of the following types of data:

|  | NA^[[2]](#footnote-2)^ | AL | A |
| --- | --- | --- | --- |
| Pharmaceutical usage and pricing | 🞎 | 🞎 | 🞎 |
| Hospital level data (e.g. utilization rates) | 🞎 | 🞎 | 🞎 |
| Health outcomes (e.g. mortality, QALYs) | 🞎 | 🞎 | 🞎 |
| Costs of service delivery (eg. salaries of health professionals) | 🞎 | 🞎 | 🞎 |
| Other, please specify ……………………… | 🞎 | 🞎 | 🞎 |

1. Please provide information on the existing HTA infrastructure in your context: *(select all that appl*y)

🞎 Methodological guidelines for HTA 🞎 Institutional processes

🞎 Database of HTA studies 🞎 Decision Criteria (threshold)

🞎 Health management information systems (HMIS)

🞎 Other, please specify…………………………………………………………………….…

1. Please identify training needs to improve HTA capacity of evidence generators *(select all that apply)*:

🞎 Introduction and Application of HTA 🞎 Topic Selection Process for HTA

🞎 Systematic Reviews 🞎 Meta-analysis

🞎 Measuring Health Outcomes 🞎 Costing Healthcare

🞎 Health Economic Evaluation & Economic Modelling

🞎 Other, please specify…………………………………………………………………….…

# Part IV: Role of your organization in HTA

1. Please provide the name of your organization:

……………………………………………………..…………………………………………

1. Please indicate the type of your organization *(select one)*:

🞎 Government (ministry, autonomous institution, others)

🞎 Private sector

🞎 Not-for-profit non-governmental organization

🞎 Research institute

🞎 Academic institute

🞎 Other, please specify…………………………………………………………………….…

1. Please provide the location (city/town/state) of your organization:

……………………………………………………..…………………………………………

1. At which level does your organization operate?

🞎 National 🞎 State

🞎 Both 🞎 Other, please specify ……………………………

1. Do you see your organization as a generator or user of HTA evidence?

🞎 Generator 🞎 User

🞎 Both 🞎 Other, please specify ……………………………

1. Please provide ONE topic on a health intervention that can potentially be an HTA research topic.

……………………………………………………..………………………………………

1. For the priority topic you provided above, please suggest at least ONE research question that you think should be examined.

Criteria for consideration: high probability of impact of research, capacity to conduct study, feasibility of study within time frame of 1.5 years.

……………………………………………………..…………………………………………

1. Please identify your organization's strengths in terms of using and/or generating evidence for HTA.

……………………………………………………..…………………………………………

1. Please identify the constraints your organization faces in terms of using and/or generating evidence for HTA.

……………………………………………………..………………………………………

1. Do you have any additional comments?

……………………………………………………..………………………………………

……………………………………………………..………………………………………

……………………………………………………..………………………………………

……………………………………………………..………………………………………

Thank you for your participation in this survey!

1. NI – Not Important, SI – Slightly Important, MI – Moderately Important, I – Important, VI – Very Important, N/A – Not Applicable [↑](#footnote-ref-1)
2. NA – Not available, AL – Available with limitation, A – Available [↑](#footnote-ref-2)
